# Clinical Trial Research on Mongolian Medical Warm Acupuncture in Treating Insomnia

**DOI:** 10.1155/2016/6190285

**Published:** 2016-12-05

**Authors:** Agula Bo, Lengge Si, Yuehong Wang, Lan Xiu, Rihan Wu, Yutang Li, Rigenjiya Mu, Latai Ga, Mei Miao, Fu Shuang, Yunhua Wu, Qiu Jin, Suocai Tong, Gerile Wuyun, Wurihan Guan, Rigen Mo, Sileng Hu, Lixia Zhang, Rui Peng, Lidao Bao

**Affiliations:** ^1^College of Traditional Mongolian Medicine, Inner Mongolia Medical University, Hohhot 010110, China; ^2^Department of Mongolian Medicine, Affiliated Hospital of Inner Mongolia University for the Nationalities, Tongliao 028000, China; ^3^Department of Mongolia Medicine, Inner Mongolia International Mongolian Hospital, Hohhot 010010, China; ^4^Department of Mongolia Medicine, Affiliated People's Hospital of Hospital of Inner Mongolia Medical University, Hohhot 010010, China; ^5^Department of Pharmacy, Affiliated Hospital of Inner Mongolia Medical University, Hohhot 010059, China

## Abstract

*Objective.* Insomnia is one of the most common sleep disorders. Hypnotics have poor long-term efficacy. Mongolian medical warm acupuncture has significant efficacy in treating insomnia. The paper evaluates the role of Mongolian medical warm acupuncture in treating insomnia by investigating the Mongolian medicine syndromes and conditions, Pittsburgh sleep quality index, and polysomnography indexes.* Method.* The patients were diagnosed in accordance with International Classification of Sleep Disorders (ICSD-2). The insomnia patients were divided into the acupuncture group (40 cases) and the estazolam group (40 cases). The patients underwent intervention of Mongolian medical warm acupuncture and estazolam. The indicators of the Mongolian medicine syndromes and conditions, Pittsburgh sleep quality index (PSQI), and polysomnography indexes (PSG) have been detected.* Result.* Based on the comparison of the Mongolian medicine syndrome scores between the warm acupuncture group and the drug treatment group, the result indicated *P* < 0.01. The clinical efficacy result showed that the effective rate (85%) in the warm acupuncture group was higher than that (70%) in the drug group. The total scores of PSQI of both groups were approximated. The sleep quality indexes of both groups decreased significantly (*P* < 0.05). The sleep quality index in the Mongolian medical warm acupuncture group decreased significantly (*P* < 0.01) and was better than that in the estazolam group. The sleep efficiency and daytime functions of the patients in the Mongolian medical warm acupuncture group improved significantly (*P* < 0.01). The sleep time was significantly extended (*P* < 0.01) in the Mongolian medical warm acupuncture group following PSG intervention. The sleep time during NREM in the Mongolian warm acupuncture group increased significantly (*P* < 0.01). The sleep time exhibited a decreasing trend during REM and it decreased significantly in the Mongolian warm acupuncture group (*P* < 0.01). The percentage of sleep time in the total sleep time during NREM3+4 in the Mongolian medical warm acupuncture group increased significantly.* Conclusion.* Mongolian medical warm acupuncture is efficient and safe in treating insomnia. It is able to better improve the patients' sleep time and daytime functions. It is better than that in the estazolam group following drug withdrawal in terms of improving the sleep time. It is more effective in helping the insomnia patients than hypnotics.

## 1. Introduction

Insomnia is a subjective experience of sleep with its quality or amount insufficient to meet the physiological needs as a result of difficulties in sleep onset and/or sleep maintenance. It can influence the social functions. It is one of the most common sleep disorders [1]. The clinical data have shown that long-term insomnia will cause increased morbidity rates of many disease such as heart diseases, hypertension, hyperlipidemia, senile dementia, depression, and anxiety. Therefore [2], the effective prevention and treatment of insomnia is still one of the global concerns [3].

Insomnia is the result of damage to the anatomical structure that controls the sleep and neurotransmitter transmission dysfunctions due to various causes [4]. Modern medicine mainly uses hypnotics. However, drug treatment is not an ideal therapeutic method due to its many side effects, poor long-term efficacy, addiction, tolerance, and so forth in the case of long-term administration [5]. In recent years, the reports on improving sleep with various therapeutic approaches in clinical application (pharmacologic and nonpharmacological) are too numerous to mention one by one. However, there is no overall research on the treatment action mechanism except for clinical efficacy. At present, seeking a safe and effective method for prevention and treatment of insomnia is one of the important topics of clinical and experimental research.

In Mongolian medicine, insomnia is also called sleep uneasiness. It is a disease characterized by frequent loss of normal sleep as a result arising from disequilibrium of “Sangen” (Sangen are the basic conditions for survival and life activities of humans) and disorders of “body, heart, and speech” functions. Disequilibrium of “Sangen,” prevalence of “Haoyi” (Haoyi is classified into five categories, i.e., Siming Haoyi, Shangxing Haoyi, Puxing Haoyi, Tiaohuo Haoyi, and Xiaqing Haoyi. They exist in the main veins, parietal regions, hearts, stomachs, and anus), and dysfunctions are the basic causes of the disease. Negative moods such as tension, worry, fear, depression, and anxiety, and social environment, diet, daily life, movement, and so forth belong to external causes. Alleviating “Haoyi” and regulating equilibrium of “Sangen” are the main treatment principles [6]. Mongolian medicine improves sleep and treats insomnia by alleviating “Haoyi,” unclogging Haoyi blood, and balancing Sangen. Warm acupuncture is one of the most common methods for treatment of insomnia in Mongolian medicine. It plays roles in unclogging meridians, regulating qi and blood and strengthening the immunity, and preventing and treating diseases [7]. It has been widely accepted by the patients as a result of its effectiveness, safety, no side effects, simplicity, no drug dependence and so forth. Therefore, the warm acupuncture has significant efficacy in treating insomnia. It is safe, simple, and economical [8].

The research involves the first clinical test of the efficacy of Mongolian medical warm acupuncture. The paper evaluates the role of Mongolian medical warm acupuncture in treating insomnia by investigating the Mongolian medicine syndromes and conditions, Pittsburgh sleep quality index, and polysomnography indexes.

## 2. Experimental Methods

### 2.1. Diagnostic Criteria

The patients were diagnosed in accordance with Standards for Diagnosis and Efficacy of TCM Symptoms and International Classification of Sleep Disorders (ICSD-2) by US Sleep Research Society [9]. The diagnostic criteria in Mongolian medicine were based on the diagnostic criteria for insomnia in the textbook of Internal Medicine of Mongolian Medicine for medical colleges and universities in China [6]. The primary symptoms include Haoyi blood and Haoyi Xila symptoms arising from prevalence of Haoyi, that is, difficulty falling asleep, easy awakening, inability to fall asleep after awakening, and pernoctation. The secondary symptoms include headache and dizziness, palpitation, amnesia, dreaminess, irritability, fatigue, and lacking in strength [10].

### 2.2. Inclusion Criteria

Inclusion criteria are as follows: (1) aged 20–75 years; (2) course of disease between 1 month and 2 years; (3) difficulty falling asleep and time for falling asleep was over 30 minutes; (4) sleep maintenance disorders, awakening times ≥ 2 overnight.

### 2.3. Criteria for Exclusion

Criteria for exclusion are as follows: (1) pregnant women and women in the lactation period; (2) patients with serious disease or in the terminal stage of diseases; (3) patients with major depressive disorders (in accordance with Chinese Classification of Mental Disorders III (CCMD-3)) (depression can be divided into mild depression and major depression based on the degree of damage to the social functions. Depression can also be divided into depression without psychotic symptoms and depression with psychotic symptoms based on the presence or absence of psychotic symptoms such as illusion, delusion, and catatonic syndrome [11]); (4) patients with severe chronic diseases (the judgment criteria were regular use of drug treatment); (5) patients with pain symptoms (such as rheumatism, rheumatoid disease, sciatica, and lumbar intervertebral disc herniation); (6) night shift workers.

### 2.4. Grouping and General Data of Patients

All cases used in the paper were hospitalized patients diagnosed as having insomnia in the Mongolian Department of the Affiliated Hospital of Inner Mongolia Medical University from September 2015 to December 2015. 80 cases were divided into 2 groups: Group 1, acupuncture group; Group 2, estazolam group using the stratified sampling random method in statistics. Each group was divided into three strata: mild, moderate, and severe based on the illness degree.

### 2.5. Intervention Method

Warm acupuncture group: special silver needles were used for treatment. Acupoint selection: selection consisted of Dinghui Acupoint, Haoyi Acupoint, and Xin Acupoint. Dinghui Acupoint: it is at the intersection between the median line of the head and the line of the tips of ears. Haoyi Acupoint: it is at the center of the superior fovea of the first thoracic vertebrae. Xin Acupoint: it is at the center of the superior fovea of the seventh thoracic vertebrae. Needles: they are Mongolian silver needles (0.5 × 30 mm, Mongolian Medicine Clinical Apparatus Research Center, Inner Mongolia Medical University, Hohhot, China). Acupuncture method: the patients were placed in a sitting position. The skin was routinely sterilized with 75% alcohol. The needle was obliquely inserted into the Dinghui Acupoint to the extent that the patients felt swelling in the scalp. The needles were inserted into the Haoyi Acupoint and Xin Acupoint. The Mongolian medicine MY-I electric heating needle warmer (Research Center of Mongolian Medicine Clinical Apparatus of Inner Mongolia Medical University) was used to warm the needles at 38°C. One Acupoint was subject to treatment for 30 minutes 4 times each day. Nine days constitutes a course of treatment. Six courses of treatment were needed and treatment was performed at an interval of 3 days.

**Figure 1:**
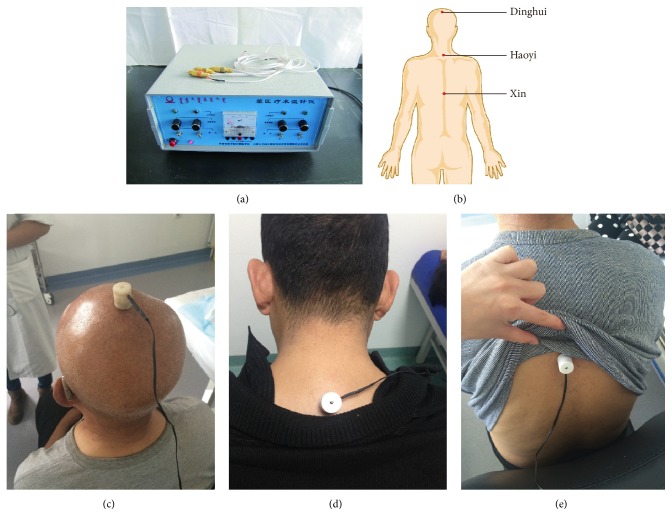
(a) Mongolian medicine MY-I electrical heating needle warmer; (b) position diagram for Dinghui Acupoint, Haoyi Acupoint, and Xin Acupoint; (c) acupuncture diagram for Dinghui Acupoint; (d) acupuncture diagram for Haoyi Acupoint; (e) acupuncture diagram for Xin Acupoint.

Drug group, estazolam (North China Pharmaceutical Co., Ltd., Batch number 152304): the dose of common usage was 1 mg. It was taken 15–30 minutes before sleep. The treatment time lasted for 56 days (see Figure 1).

### 2.6. Observation Index

#### 2.6.1. Observation of Mongolian Medicine Indicators

The Mongolian medicine syndromes and conditions were judged on the basis of scores of Mongolian medicine syndromes and conditions and by reference to Internal Medicine of Mongolian Medicine.

The scores were as follows: mild, less than 5 points; moderate, 6 points to 10 points; severe, 11 points to 15 points.

#### 2.6.2. Pittsburgh Sleep Quality Index (PSQI) Scale

The Pittsburgh sleep quality index was used to assess the sleep quality of the subjects in recent 1 month. The full mark of the scale was 21 points. Judgment criteria for efficacy: A ratio of the scores before treatment minus the scores after treatment to the scores before treatment × 100% <30% indicated that there was no efficacy. A ratio ≥ 30% indicated that there was efficacy. A ratio ≥ 70% there was significant efficacy. A ratio ≥ 90% indicated that the patients made recovery [12].

#### 2.6.3. Detection of Polysomnography Indexes (PSG)

The Embletta 13 (X100) lead portable polysomnography system (Medcare, Greensburg, PA, USA) was used to analyze the sleep structure. The indexes included total sleep time, awakening time, movement awakening time, awakening times, NREM, REM sleep time, and NREM S3∖S4 time. These indexes were measured before warm acupuncture and drug intervention and measured again 7 days after warm acupuncture and drug intervention [13].

### 2.7. Statistical Analysis Method

The SPSS18.0 statistical software was used to analyze all data. The paired* t*-test was used to analyze the changes in patients in each group before and after treatment. The difference was considered to be significant when *P* < 0.05. The experimental data were expressed by mean ± standard deviation. Student's* t*-test was used to detect the difference between the two groups. The comparison of the intergroup enumeration data used chi-square test or Wilcoxon signed rank test. The difference was considered to be significant when *P* < 0.05. The difference was considered to be statistically significant when *P* < 0.01.

## 3. Result

### 3.1. General Data of Patients

There were 80 cases included in the paper. The Mongolian medical warm acupuncture group had 40 cases and the estazolam group had 40 cases. Group 1 was the Mongolian medical warm acupuncture group with 4 mild cases, 22 moderate cases, and 14 severe cases, 40 cases in total, 24 males and 16 females aged 21–65 years, age range 43.25 ± 9.56 years, and range of course of disease 3.15 ± 1.24 years. Group 2 was the estazolam group with 4 mild cases, 19 moderate cases, and 17 severe cases: 40 cases in total, 22 males and 18 females, aged 19–67 years, aged 47.21 ± 8.31 years on average, and average range of course of disease 4.10 ± 1.06 years. The result was shown in Table 1.

### 3.2. Indexes of Mongolian Medicine Syndromes and Conditions

There was no significant difference in scores of Mongolian medicine syndromes between the two groups based on* t*-test before treatment (*P* > 0.05), indicating that they were comparable. The* t*-test indicated that there was no significant difference between the two groups after treatment (*P* > 0.05), indicating that they were comparable. The comparison between the warm acupuncture group and the drug treatment group indicated that there was an extremely significant difference in scores of Mongolian medicine symptoms. The result was shown in Table 2.

### 3.3. Analysis of Clinical Efficacy Results

The efficacy result was judged using the score method. The full mark was 21 points. Efficacy judgment is ratio of difference of the score before treatment minus the score after treatment to the score before treatment × 100%: (1) recovery ≥ 9096; (2) significant efficacy ≥ 70%; (3) efficacy ≥ 3096; (4) no efficacy < 30%. The result was shown in Table 3.

### 3.4. Evaluation of Pittsburgh Sleep Quality Index

**Figure 2:**
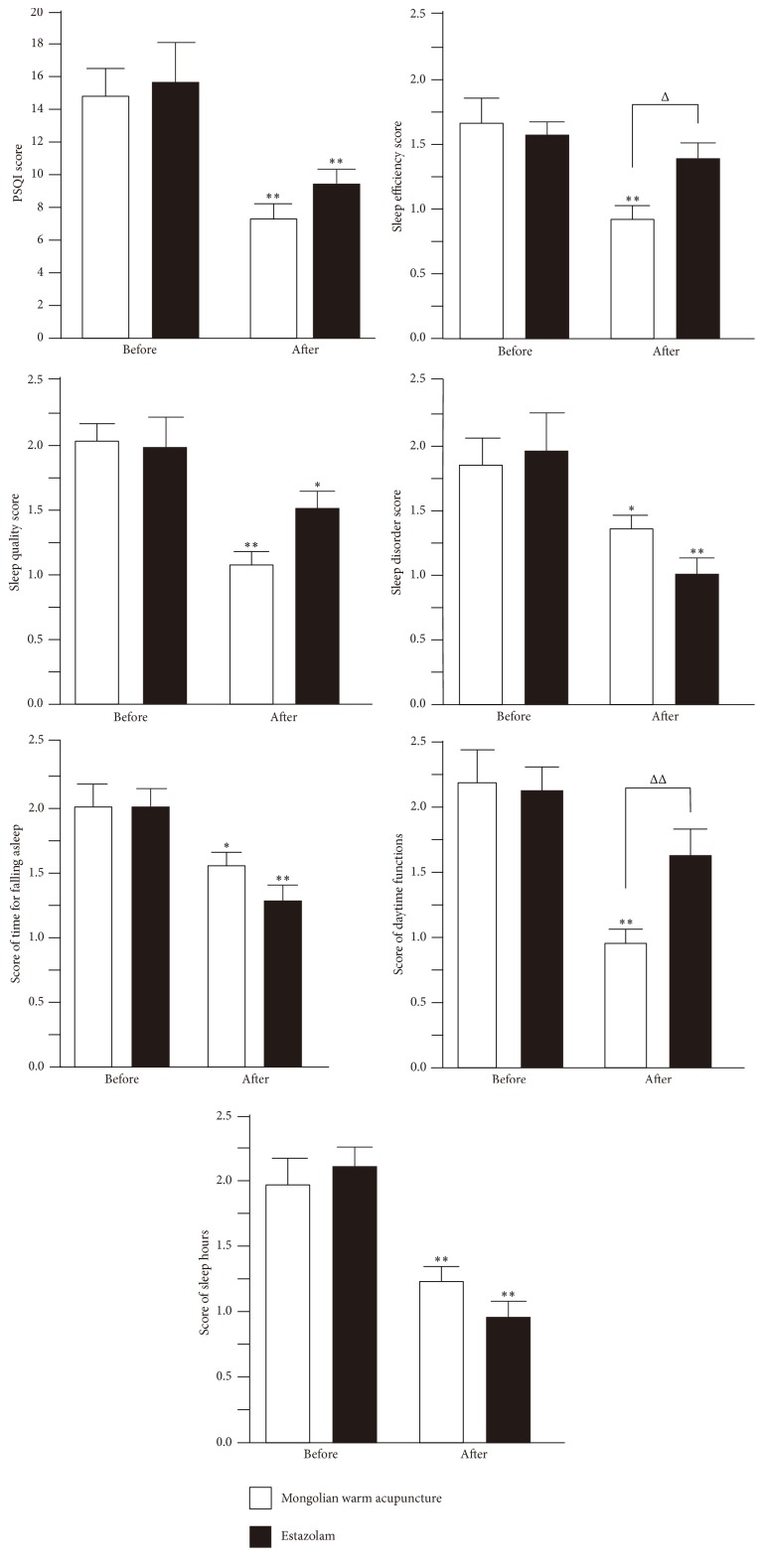
Note: *∗* indicated that there was a significant difference between the numerical value before treatment and that after treatment; *∗* indicated *P* > 0.01. *∗∗* indicated *P* > 0.01. Δ indicated that there was a significant difference in numerical value between the Mongolian medical warm acupuncture group and the estazolam group. Δ indicated *P* > 0.01; ΔΔ indicated *P* > 0.01.

The total scores of PSQI in both groups before treatment were close. The* t*-test indicated that *t* = −0.314 and *P* = 0.847. There was no significant difference in Pittsburgh sleep quality index between the two groups, indicating both groups were comparable. The sleep quality indexes in both groups decreased significantly after treatment (*P* > 0.05). The treatment was considered to be efficacious. The score in the Mongolian medical warm acupuncture group was slightly lower but there was no significant difference between both groups (*P* > 0.05). The sleep quality index in the Mongolian medical warm acupuncture group improved significantly after treatment (*P* > 0.01), which was better than that in the estazolam group (*P* > 0.01). The time for falling asleep decreased significantly (*P* > 0.05); the sleep time increased significantly (*P* > 0.01); the sleep disorder score decreased significantly (*P* > 0.05). However, the three indexes indicated that the therapeutic effect was not as good as that in the estazolam group. The sleep efficiency and daytime functions of the patients in the Mongolian medical warm acupuncture group improved significantly after treatment (*P* > 0.01). The sleep efficiency and daytime functions in the estazolam group also improved significantly (*P* > 0.01). The result was shown in Figure 2.

### 3.5. Detection of Polysomnography Indexes (PSG)

**Figure 3:**
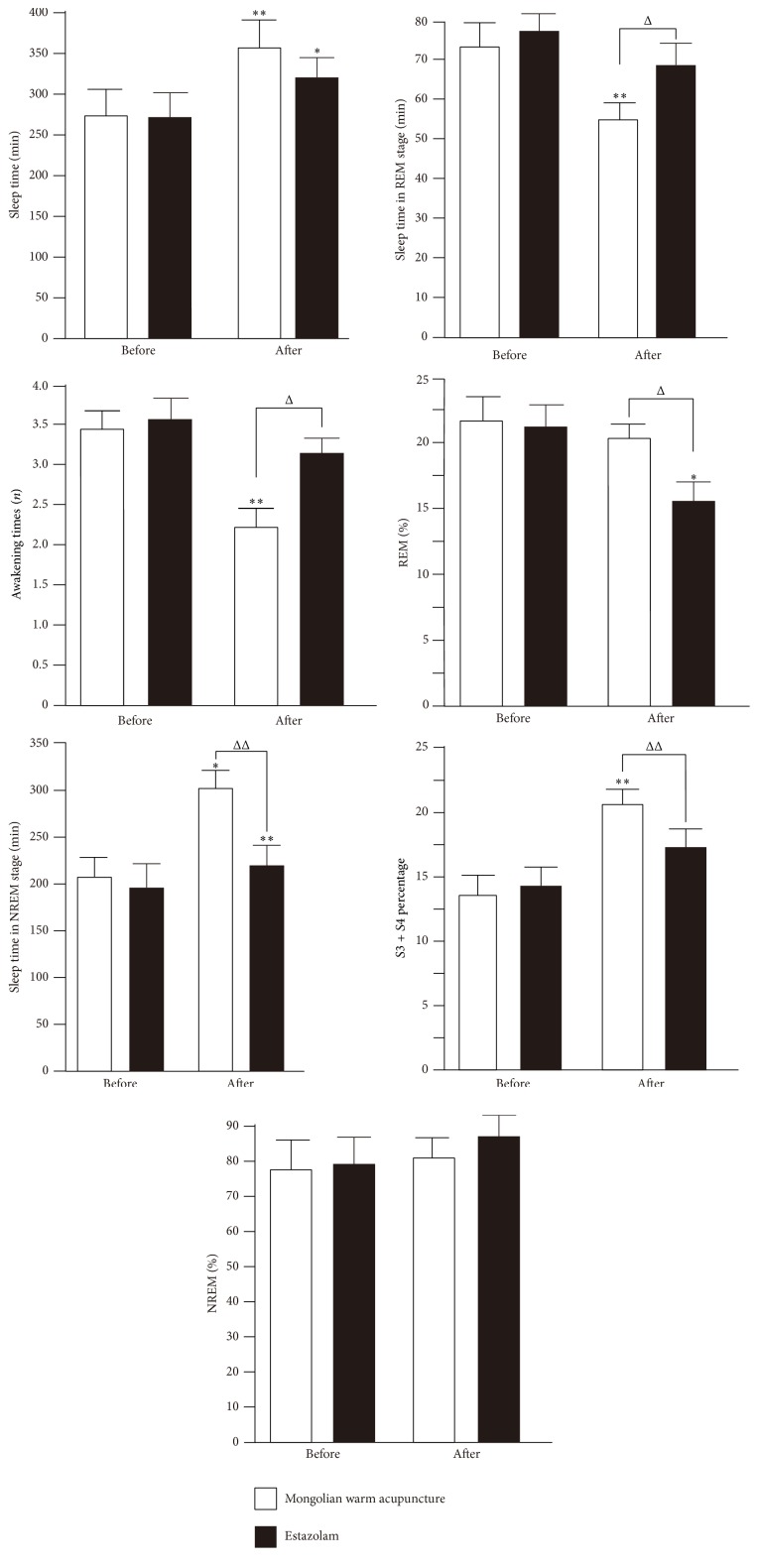
Note: *∗* indicated that there was a significant difference between the numerical value before treatment and that after treatment; *∗* indicated *P* > 0.01. *∗∗* indicated *P* > 0.01. Δ indicated that there was a significant difference in numerical value between the Mongolian medical warm acupuncture group and the estazolam group. Δ indicated *P* > 0.01; ΔΔ indicated *P* > 0.01.

The mean value of all polysomnography indexes of the patients in both groups was subject to* t*-test before treatment. *P* > 0.05 indicated that the two groups were comparable. The total sleep time in both groups exhibited a significant increasing trend after treatment. The sleep time in the Mongolian medical warm acupuncture group extended significantly (*P* > 0.01). The nighttime awakening times decreased significantly only in the Mongolian medical warm acupuncture group. The patients still awakened frequently after drug withdrawal in the estazolam group. There was a significant difference between both groups (*P* > 0.01). The sleep time during the NREM period increased significantly in the Mongolian medical warm acupuncture group when compared with that in the estazolam group before treatment or after treatment (*P* > 0.01). No significant changes in percentage of sleep time during the NREM period occurred in both groups before and after treatment. The sleep time during the NREM period exhibited a decreasing trend and the sleep time in the Mongolian medical warm acupuncture group exhibited a significant decreasing trend (*P* > 0.01). The percentages of the sleep time during the REM period in the total sleep time in both groups exhibited a decreasing trend and the percentage in the estazolam group exhibited a significant decreasing trend (*P* > 0.05). The percentages of the sleep time during the NREM3+4 period in the total sleep time increased in both groups before and after treatment and the percentage in the Mongolian medical warm acupuncture group increased significantly (*P* > 0.01). It also increased significantly when compared with that in the estazolam group. The treatment was considered to be efficacious. The result was shown in Figure 3.

## 4. Discussion

Insomnia is a significant public health issue with a high morbidity. Insomnia may finally lead to psychological, physiological, and occupational health issues if no therapy is administered [14]. There are many related factors resulting in insomnia, such as physiological-psychological factors, genes, organism quality, environmental conditions, social interpersonal relationship, mental stimulation, cognitive process, body disease, mental diseases, adverse drug reactions, and sleeping habits. The pathogenic mechanisms of insomnia are complex and many of them still remain unclear [15]. Drug treatment is still one of the main methods for treatment of insomnia both at home and abroad [16]. The current medicines for treatment of insomnia are mainly specific to the receptor of *γ* gamma amino acid butyric acid (GABA), melatonin receptor, histamine receptor, orexin and serotonin receptors [17]. The medicines include the first-generation barbiturates, the second-generation benzodiazepines, and the third-generation benzodiazepines [18].

Many patients prefer nonmedicine treatment as orally administered drugs have a short duration of efficacy and long-term medication has drug dependence and drug withdrawal reactions [19]. In the Mongolian medical warm acupuncture therapy, special silver needles were used to stimulate the fixed sites or other sites of the human body for prevention and treatment of diseases by acupuncture and warming moxibustion. It produces some biological effects for treatment of diseases by means of acupuncture effect, warm effect, and specific stimulation of acupoints. A great deal of clinical research has shown that Mongolian medical warm acupuncture is simple, efficient, and safe in treating insomnia. In Mongolian medicine, it is considered that the warm needling acupuncture plays roles in dredging meridians, regulating qi and blood, and strengthening the immunity. Modern research has demonstrated that acupuncture plays roles in regulating the neurohumour, strengthening, activating, and mobilizing the disease resistance, and improving the immunity of the organism. It is a good choice for insomnia patients.

Based on the clinical test, Mongolian medical warm acupuncture is able to better improve the patients' sleep time and daytime functions. It is better than that in the estazolam group following drug withdrawal in term of improving the sleep time. It is more effective in helping the insomnia patients than hypnotics.

As a disease entity, the correlation between insomnia and electroencephalogram has aroused extensive attention. In foreign literature, it is further considered that a decreased REM latency period is the characteristic change in insomnia. In addition, there are such abnormalities as decreased S3 and S4 sleep, increased REM density, decreased sleep efficiency, and so forth [20]. The characteristic changes of insomnia PSG are the change in sleep continuity and abnormality in structure including increased awakening times at night, decreased actual sleep time, and low sleep efficiency. Imbalance occurs to the sleep structural proportion. The percentage of sleep at stage S1 and stage S2 (light sleep) in the total sleep stage increases and the deep sleep decreases significantly [21]. The data in the test before intervention have demonstrated the above opinion.

Mongolian medical warm acupuncture can decrease the awakening times, the sleep hours in the REM period, and the REM sleep percentage and increase the NREM sleep hours. Meanwhile, the sleep quality, time for falling asleep, sleep hours, sleep efficiency, sleep disorders, daytime functions, and total scores improve significantly when compared with those before treatment. The Mongolian medical warm acupuncture group is better than the estazolam group in these indexes.

Long-term warm acupuncture treatment for the patients with significantly changed sleep physiological indexes of insomnia is able to bring some sleep indexes back to normal, such as REM sleep hours, sleep efficiency, and awakening time. Meanwhile, it plays a role in improving the characteristics indexes of insomnia, such as NREM3+4 stage. It is better than the current hypnotic treatment to a certain extent. The experiment provides clinical research verification for modern treatment with Mongolian medical warm acupuncture and scientific support for modernization development of the traditional ethnic medicine [22].

## Figures and Tables

**Table 1:** General data of patients.

Group	Number of cases	Sex		Course of disease (years)	Age (years)	Condition		
		Male	Female			Mild	Moderate	Severe
Warm acupuncture group	40	24	16	3.15 ± 1.24	43.25 ± 9.56	4	22	14
Drug group	40	22	18	4.10 ± 1.06	47.21 ± 8.31	4	19	17
		*χ* ^2^ = 0.292		*t* = 0.651	*t* = 0.717	*z* = −1.303		
		*P* = 0.587		*P* = 0.534	*P* = 0.536	*P* = 0.319		

The gender comparison between both groups used chi-square test. The comparisons of course of disease and age used *t*-test. The comparisons of conditions used the rank sum test. There were statistical differences in statistical indexes such as age, course of disease, and conditions. The conditions were evaluated. Rank coding was performed in the rank sum test (mild: 1; moderate: 2; severe: 3). The Mongolian medical warm acupuncture group and the estazolam control group were subject to a rank sum test (*z* = −1.303, *P* = 0.319). There was no statistical difference in conditions between the two groups (*P* > 0.05).

**Table 2:** Indexes of Mongolian medicine syndromes and conditions.

Group	Number of cases	Scores before treatment	Scores after treatment	*t*	*P*
Warm acupuncture group	40	11.56 ± 3.64	3.64 ± 1.08	6.302	0.000
Drug group	40	12.27 ± 4.25	4.25 ± 1.69	10.524	0.000
		*t* = 0.647	*t* = 0.631		
		*P* = 0.421	*P* = 0.596		

**Table 3:** Analysis of clinical efficacy results.

Group	Number of cases	Recovery	Significant efficacy	Efficacy	No efficacy	Effective rate
Warm acupuncture group	40	2	18	14	6	85%
Drug group	40	0	13	15	12	70%
